# Extraction of pigments from camellia seed husks and their application on silk fabrics

**DOI:** 10.1039/d2ra06793e

**Published:** 2022-12-02

**Authors:** Jie Chen, Yu Ni, Bohao Mei, Huiyu Jiang, Yunli Wang, Yangyi Chen, Huan Qi

**Affiliations:** College of Chemistry and Chemical Engineering, Wuhan Textile University Wuhan 430200 China; College of Textiles and Apparel, Quanzhou Normal University Fujian 362002 China qhqh123@126.com

## Abstract

To reuse camellia husk waste and expand the scope of natural dyes, this research proposes pigment extraction from camellia husks and investigates various properties when applied on silk fabrics. Single-factor experiments were used to screen and optimize the dyeing process. The extracted pigments and dyed fibers were analyzed and characterized by LC-MS, FTIR and SEM, respectively. Six metal mordants were compared with each other, and their possible mordanting mechanisms were proposed. Color fastness, UV resistance, and antioxidant and antibacterial properties were evaluated after dyeing. The results showed that the optimal dyeing process was as follows: dyestuff mass 50 g L^−1^, holding time 45 min, bath pH 3.0, holding temperature 100 °C. LC-MS and FTIR results showed that the pigments in the extracts were mainly dimeric and multimeric procyanidins. Metal ion addition increased the *K*/*S* value while the pre-mordanting method had a superior dyeing depth. The rubbing and washing fastness of the dyed fabric were all above grade 4.0. Meanwhile, the dyed sample exhibited favorable UV resistance, and antioxidant and antibacterial properties, including a UPF index of 63.4 and an inhibition rate of 98.74% and 97.39% for *S. aureus* and *E. coli*, respectively.

## Abstract 2

The camellia husk natural dye extraction process.

## Graphical abstract

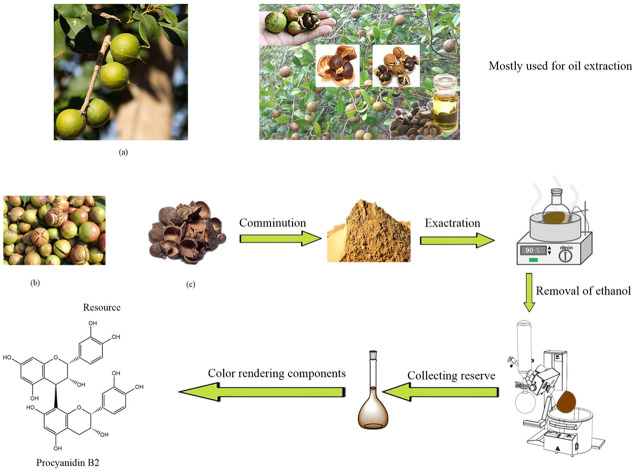

## 1. Introduction

With the rapid development of the textile industry and growing environmental concerns, natural dyes have received renewed attention and are being used in industry.^1^ Pigments derived from natural resources are considered to be eco-friendly dyes with good biocompatibility and rapid degradability in natural environments.^2^ In addition, many natural dyes have companion components capable of imparting certain specific functions to textiles, such as UV resistance,^3^ antibacterial,^4^ antiviral,^5^ antioxidant,^6^ and flame-retardant properties.^7^ In recent years, the application of natural dyestuffs on textiles has attracted research interest, but there are many problems: scarce pigment sources, low staining rate, poor fastness and incomplete colour distribution, which cannot meet the requirements of industrial applications. Therefore, it is important to develop more pigment sources and improve the dyeing properties for the industrial application of natural dyestuffs.

Silk has a long history of development in human society, and is highly respected for its good mechanical properties, polished and elegant clothing qualities and excellent taking properties.^7,8^ Nevertheless, silk fibres are by no means without limitations. Silk suffers from a number of drawbacks such as wrinkling, photo-oxidation, deterioration, yellowing, poor UV protection, poor antioxidant capability, poor antibacterial activity and so on.^9–12^ These deficiencies inevitably limit the application of silk fibres. Therefore, measures must be taken to overcome these shortcomings and improve the functionality of silk.^13^

Camellia sinensis is a small evergreen broad-leaved tree and widely distributed in certain alpine areas in Asia. Camellia sinensis fruit can be extracted for oil, which is a high-grade natural oil unique to the region. Camellia husk was the shell of its seeds removed before oil extraction, which contains camellia saponins, camellia seed proteins, camellia seed polysaccharides and so on.^14^ For a long time, camellia husks were used for crop composting and fuels with inefficient utilization.^15^ Therefore, it is important to investigate the reuse technology to realize the comprehensive utilization. Procyanidins are a general term for polyphenolic compounds that are widely found in the hull and bast of plants, and are also the main components of plant pigments. There are many reports on the extraction of polyphenols and their derivatives from plants for textile dyeing, such as polyphenolics extracted from Buddleja officinalis, flora leaves, Cacao pod husk, black tea, *etc.*, which can obtain superior dyeing performance on fibres. Procyanidin has been applied to metal coatings and the treated surface exhibits excellent corrosion resistance and antibacterial property.^16^ The main pigment component of camellia husks extract is procyanidin and its derivates.^17,18^ The extraction of dyes from waste green tea leaves and their use in cotton fabric dyeing by Sukemi *et al.*^19^ However, the procyanidin extracted from waste camellia husks and applied for silk dyeing were rarely reported. The functionality of procyanidin dyed fabrics needs to be thoroughly investigated. It will be a practical guidance for the full recycling of waste camellia husks.

In this work, the pigments were extracted from camellia husk and applied for silk dyeing. The single-factor experiment was selected to optimize the dyeing process. The chemical components of extract were analysed by Fourier transform infrared spectroscopy (FTIR) and liquid chromatography-mass spectrometry (LC-MS). Morphological changes on fibre surface after dyeing were recorded on scanning electron microscope (SEM). The effect of different metal salt mordanting conditions on the dyeing effect was compared. The colour fastnesses, ultraviolet resistance, antioxidant and antibacterial performances of dyed fabric were tested. These results provided a reference for the reuse of waste camellia husks and the enrichment of the source range of natural dyes.

## 2. Materials and methods

### 2.1. Materials

Camellia husks were purchased from an oil extraction plant in Quanzhou, China. Silk fabric was purchased from the local market in Wuhan (warp-knitted fabric, 20 g m^−2^). Acetic acid, sodium chloride, sodium hydroxide, anhydrous ethanol, methanol, ferrous sulphate heptahydrate, copper sulphate pentahydrate, aluminium sulphate octadecahydrate were supplied by Xilong Science Co. Ltd. Chromium trichloride and neodymium chloride were purchased from Shanghai Aladdin Biochemical Technology Co. Ferric chloride was supplied by Tianjin Fuchen Chemical Reagent Co. Ltd. 1,1-Diphenyl-2-trinitrophenylhydrazine (DPPH) was purchased from Hefei BOSF Biotechnology Co. Ltd. All reagents were used as received without any further purification.

### 2.2. Preparation of dye solution

**Scheme 1:**
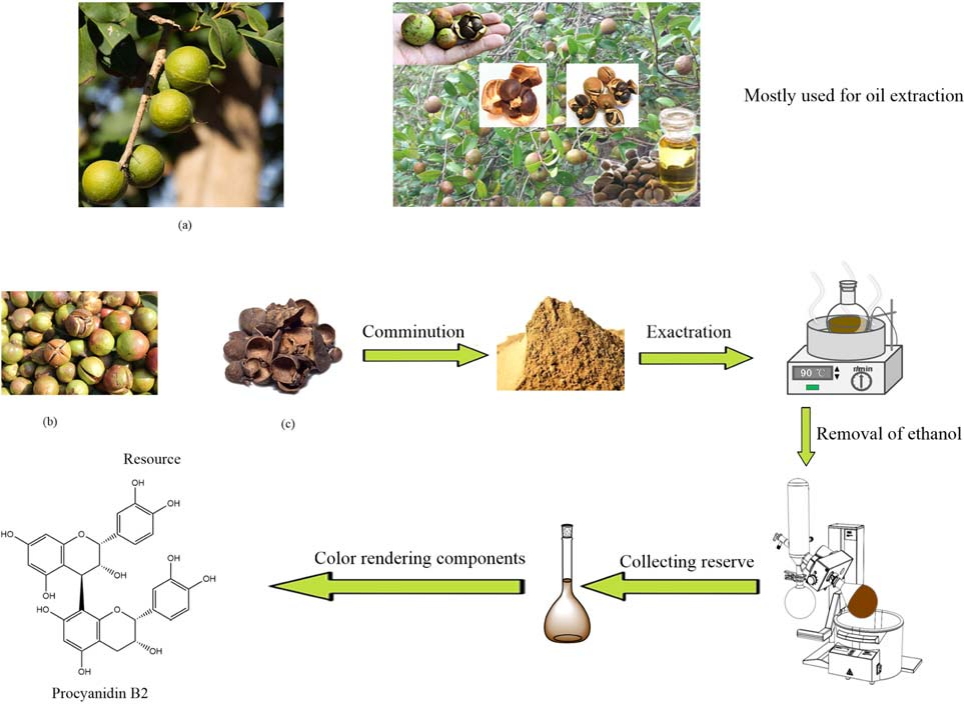
The camellia husks natural dye extraction process.

The process of preparing camellia husks dye is shown in Scheme 1. Camellia husks were weighed and crushed, collected in a flask, and then 50% ethanol was added at the ratio of 1 : 25. After that the solution was boiled and refluxed at 90 °C for 1 hour. The suspension was filtered and the filtrate was collected. After concentrating the filtrate through a rotary evaporator, it was dried in a freeze dryer to obtain a powder pigment with a 5.83% yield.

### 2.3. Single-factor experiment

The dyeing process of the control group was set as follows: dyestuff mass of 30 g L^−1^, holding time 45 min, bath pH 5.0, holding temperature 80 °C, bath ratio 1 : 30. The effects of dyestuff mass, holding temperature, holding time and bath pH on *K*/*S* value were analysed by the single-factor experiment method. The dyestuff mass was from 5 to 80 g L^−1^, the holding temperature from 50 to 100 °C, the holding time from 15 to 90 min, and the bath pH ranged from 2.5 to 10.0. After dyeing, the silk fabric was washed with tap water and dried for the test.

### 2.4. Mordant dyeing

**Scheme 2:**
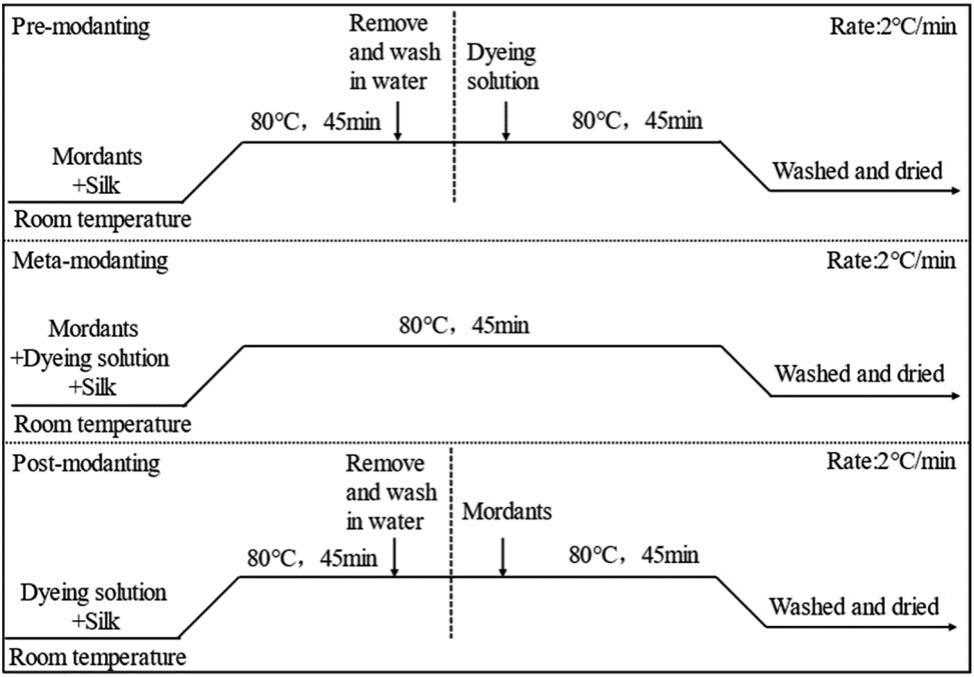
The mordant dyeing process curve.

The silk fabrics were mordant dyed with Nd^3+^, Fe^2+^, Cu^2+^, Al^3+^, Cr^3+^ and Fe^3+^ at a mordant concentration of 2.0% (On the Mass of Fabric (o.m.f.)). The dyeing process curve of the mordant was shown in Scheme 2.

### 2.5. Performance testing

#### 2.5.1. Colour characteristics

The colour characteristics of the dyed fabric were evaluated by the reflectance method on a Datecolor 800 colorimeter. The CIE laboratory colour coordinates L*, a* and b* represent the luminance, the red–green colour and the yellow–blue colour, respectively. The colour characteristic (*K*/*S*) values were calculated from the Kubelka–Monk equation (eqn (1)).

1 *K*/*S* = (1 − *R*)^2^/2*R*

where *R* is the reflectance of fabric, *K* is the absorption coefficient, and *S* is the scattering coefficient.

#### 2.5.2. Colour fastness properties

Rubbing colour fastness was tested with reference to ISO 105-X16-2001, washing colour fastness was referred to ISO 105-C10-2006, the degree of colour change was determined with reference to ISO 105-A02-1993, and the degree of staining was determined with reference to ISO 105-A03-1993.

#### 2.5.3. UV protection properties

The coloured silk fabric was folded into three layers to determine its UV protection performance on the UV protection tester (YG(B)912, China), the standard refers to GB/T 18830-2009 determination, record UVA, UVB and UPF values.

#### 2.5.4. Antioxidant activities

2 mg of DPPH dissolved in methanol solution and set aside to 50 ml. The absorbance of DPPH solution at 517 nm was recorded as *A*_1_. 10 ml of DPPH solution mixed with 30 mg fabric and retained for 30 minutes and then the absorbance at 517 nm was recorded as *A*_2_. Antioxidant activity (*J*) was calculated according to eqn (2).

2 *J* = (*A*_1_ − *A*_2_)/*A*_1_ × 100%

#### 2.5.5. Antibacterial performance

The antibacterial property of samples against *S. aureus* (*Staphylococcus aureus*) and *E. coli* (*Escherichia coli*) was carried out according to the antibacterial standard for textiles (GB/T 20944.3-2008). The specific experimental procedure and the calculation of the bacteriostatic rate were in accordance with the reference.^20^

### 2.6. Characterization

#### 2.6.1. UV-vis absorption spectroscopy

2 ml of 50 g L^−1^ camellia husks extract was diluted 50 times and the absorbance was measured from 240 nm to 400 nm on a UV-vis spectrophotometer (PERSEE TU-1950, China) and the maximum absorption wavelength was recorded.

#### 2.6.2. LC-MS analysis

Camellia husks extract was recorded and analysed by Liquid Chromatography-Mass Spectrometry. Using an Ultimate 3000 LC (column. Hypersil GOLD 100 × 2.1 mm, 3 μm Thermo Scientific, *T* = 251, wavelength: 190–400 nm, *U* = 0.25 ml min^−1^, mobile phase: A: methanol B: 0.1% formic acid solution) and a Thermo Scientific Q Exactive mass spectrometer (spray voltage: 3200 V) with gradient elution. *T* = 0-10-15-15.1-22, A% = 15-90-90-15-15) and Thermo Scientific Q Exactive mass spectrometer (spray voltage: 3200 V, capillary temperature. 300.00, intrathecal gas: 40.00 Arb, auxiliary gas: 8.00 Arb, maximum spray current.

#### 2.6.3. Morphology

The surface morphology changes of silk before and after dyeing were performed on Scanning Electron Microscopy (SEM, JSM IT500A, Japan). Before characterization, the samples were coated with platinum sputtering for 60 s.

#### 2.6.4. FTIR analysis

The nature dye powder was obtained by a cold lyophilizer from camellia husks extract. The fabric samples were cut into powder and mixed with KBr. About 2.0 mg of the powder was mixed with 200.0 mg KBr and prepared in pellet form for FTIR testing. The chemical composition of samples was recorded on a Fourier transform infrared spectrometer (PerkinElmer System 2000, Germany) in absorbance mode with a range of 4000–400 cm^−1^ and a resolution of 8 cm^−1^.

## 3. Results and discussions

### 3.1. Characterization of extracts

**Fig. 1:**
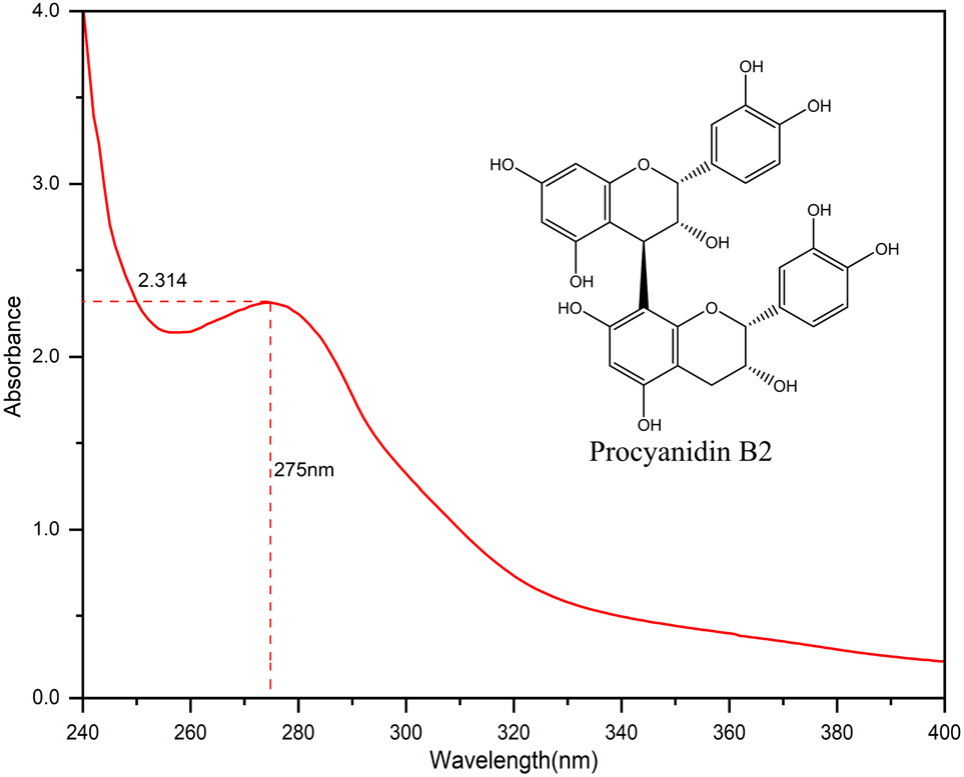
The UV-vis absorption spectrum of camellia husks extract.

**Fig. 2:**
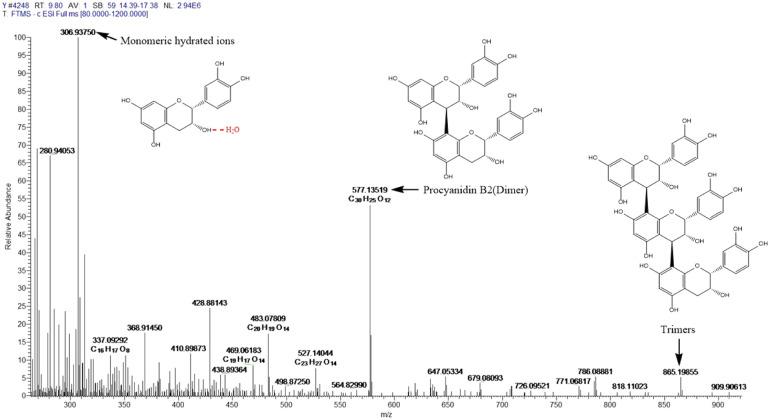
The mass spectrometry of camellia husks extract.

The UV-vis absorption spectrum of camellia husks extract is shown in Fig. 1. It is clear that the maximum absorption wavelength of the solution is 275 nm. This result indicates that camellia husks extract has the ability to absorb UV light and may have some UV protection properties. The main component of camellia husks extract that can be used for staining is procyanidin.^17,18^ Therefore, LC-MS analysis was performed on the extractive. According to the effluent peaks, the main signal peak at 7.5 min was subjected to Mass Spectrometry Analysis and the results were illustrated in Fig. 2. In the mass (*m*/*z*) range from 300 to 900, peaks apparently at 577.1 (M − H^+^) and 865.2 (M − H^+^) are corresponding to the dimers (procyanidin B2) and trimers of catechin, respectively, revealing that main components of procyanidin of extractive were dimeric and trimeric forms of catechins. Moreover, the peak at 306.9 (M − H^+^) was the catechin monomer incorporated by a molecule of water.^21^ The states of procyanidins polymerization determines its adsorption and dyeing properties on fibres.

### 3.2. Optimization of the silk dyeing process

**Fig. 3:**
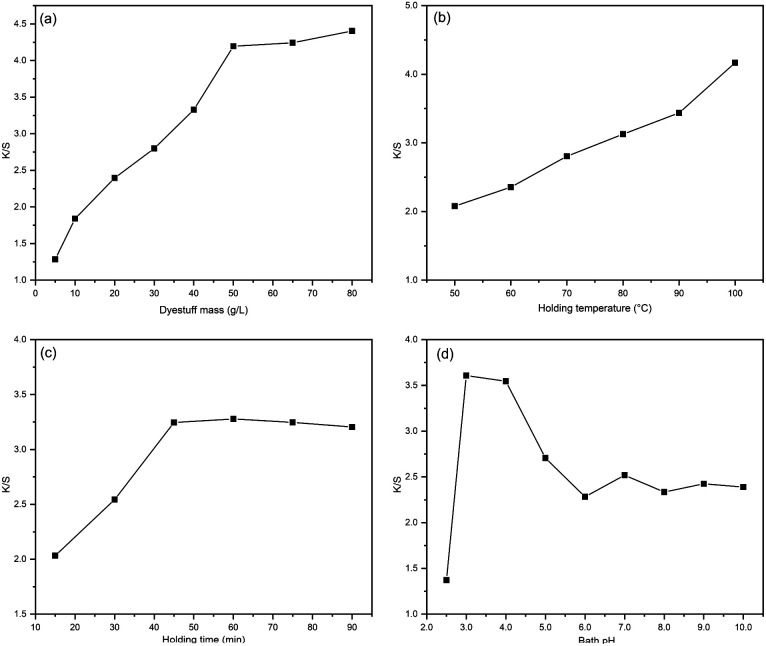
The factors effect on *K*/*S* value: (a) dyestuff mass, (b) holding temperature, (c) holding time, (d) bath pH.

The *K*/*S* value was employed as an index to evaluate the fabric's apparent colour yield. The effects of dyestuff mass, bath pH, holding temperature and time were investigated for dyeing silk fabrics. The results are summarized in Fig. 3. From Fig. 3(a), the *K*/*S* value was raised with the increasing dyestuff mass. When the concentration exceeded 50 g L^−1^, the increasing trend slowed down significantly. This is due to the fact that when the dye dosage exceeds 50 g L^−1^, the dye in the fibre becomes saturated, at which point increasing the dye mass has less effect on the colour depth and the *K*/*S* value.

As the holding temperature rises, the fabric colour yield rises significantly as shown in Fig. 3(b). High-temperature accelerates the movement and diffusion rate of the dyes as well as accelerates the hygroscopic swelling of the silk fibres, all of which are conducive to the dyeing process. Considering the high-temperature conditions will cause damage to silk fibres, so silk fabric dyeing temperature generally does not exceed 100 °C.

In Fig. 3(c), the extended holding time is conducive for the dye to diffuse fully into the fibre. When the holding time exceeds 45 minutes, the *K*/*S* value tends to be constant and the dye on fibre reaches saturation.

From the results in Fig. 3(d), the pH of the bath solution obviously affected the colour yield of the fabric, and the sample obtained the maximum *K*/*S* at pH 3.0. Under specific pH conditions, procyanidins exhibit varying dyeing mechanisms due to the different nature charges carried by the fibres and dyes, which affect the final colour depth. The isoelectric point of silk is between 3.5 and 5.2, while the phenolics in procyanidin can ionize to form phenoxy negative ions at a certain pH value. When the bath pH is higher than isoelectric point (pH > 5.2), both the fibre and the dye are negatively charged, and the electrostatic repulsion between molecules is not conducive to dyeing, resulting in low dyeing rate. When it is in the isoelectric point range (3.5 < pH < 5.2), there are two ways of bonding between fibre and dye: the terminal amino, amide and carboxyl groups on the fibre are ionized, at which point there is both electrostatic gravitational force and electrostatic repulsion between the fibre and the dye. The other part of the group on the fibre is not significantly ionized, and the fibre and dye act mainly by hydrogen bonding. When the pH of the dye bath is below the isoelectric point, the dye is fixed with the terminal amino and amide groups on the fibre in the form of ionic bonds, and with the carboxyl groups by hydrogen bonding. However, as the pH decreases further, the phenolic structure in the procyanidins changes, leading to a significant weakening of the electrostatic gravitational effect between the dye and fibre.^2^

In summary, the optimized process conditions for dyeing silk fabrics with camellia husks extract can be summarized as follows: dyestuff mass 50 g L^−1^, holding time 45 min, bath pH 3.0, holding temperature 100 °C.

### 3.3. Morphology of dyed fabric

**Fig. 4:**
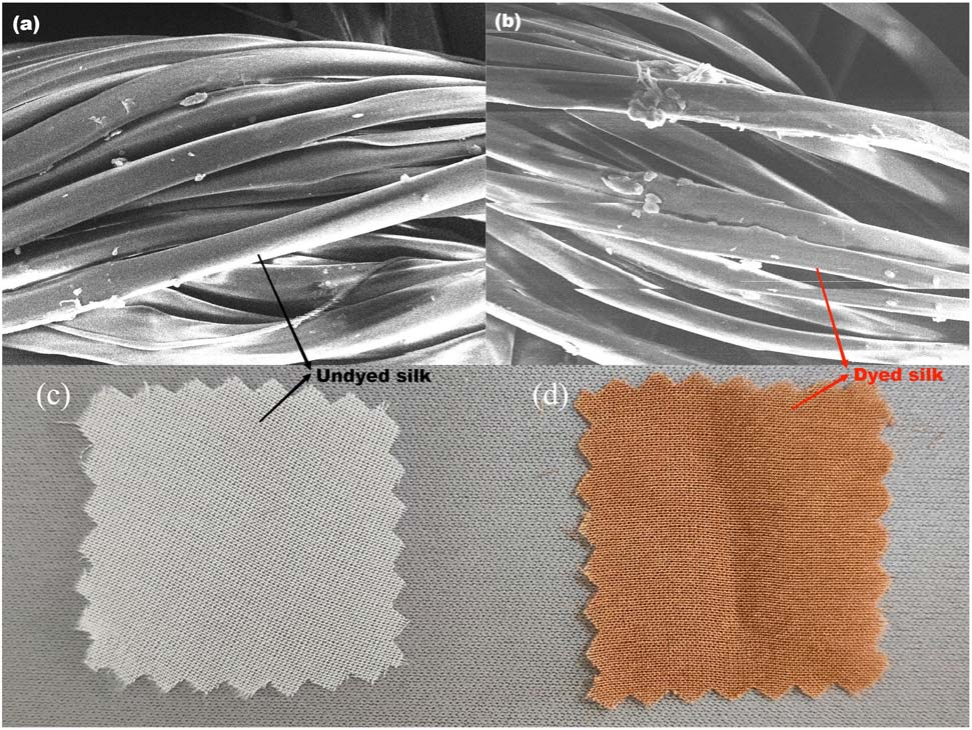
The SEM photographs of silk fibres and optical photographs of silk fabrics: (a) raw silk fibre, (b) dyed silk fibre, (c) raw silk fabric, (d) dyed silk fabric.

The effect of dyeing temperature on fibre morphology is shown in Fig. 4. From the results, it can be seen that there was no dye coating deposition on the surface of the fibres after dyeing, and the main structure of silk fibre was well preserved after high-temperature dyeing, and no significant destruction of the fibre's apparent morphology was observed. However, it was not possible to determine whether the high-temperature dyeing had caused intrinsic damage to the silk fabric, resulting in a hand feel deterioration.

### 3.4. FTIR analysis

**Fig. 5:**
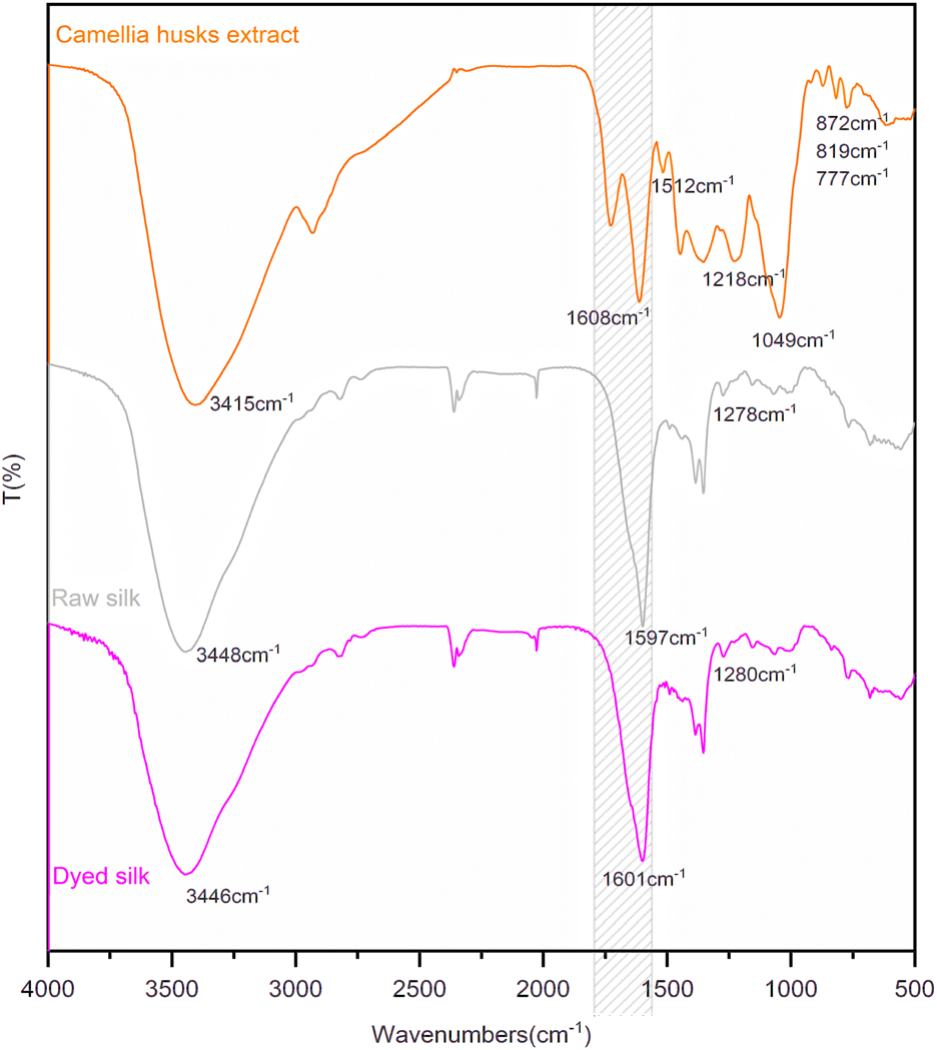
The FTIR analysis of silk fabric and pigments from camellia husks.

The infrared analysis of silk fibre is presented in Fig. 5. In FTIR spectra of the extract, 1608 cm^−1^ and 1512 cm^−1^ are the C=C stretching vibration of the benzene ring in procyanidin B2. 1218 cm^−1^ is the stretching vibration peak of the C–O bond in phenolic hydroxyl. 1049 cm^−1^ is the asymmetric stretching vibration of ether bond C–O–C. 777 cm^−1^, 817 cm^−1^ and 872 cm^−1^ are the absorption peaks of the C–H bond on the multi-substituted benzene ring, respectively.^22^ In the infrared spectrum of silk fabric, 1597 cm^−1^ is the conjunction and overlapping peak of the C=O stretching vibration peak and the deformation vibration peak in the N–H plane of the amide group. 1278 cm^−1^ is the C–N stretching vibration of amide.^22,23^ After dyeing, the position of the spectral signal changed slightly. However, the signal of the conjunction peak shifted from 1597 cm^−1^ to 1601 cm^−1^ and was significantly broadened, indicating the introduction of new groups on the fibre after dyeing. This is due to the dyeing process where the dye amount on fibre is low and its structural groups are small relative to the number of characteristic groups on fibres, which are not highlighted in the infrared absorption.

### 3.5. Mordant dyeing

#### 3.5.1. Effect of mordanting

Six metal ions Nd^3+^, Fe^2+^, Cu^2+^, Al^3+^, Cr^3+^ and Fe^3+^ were used with a mordant concentration of 2.0% (o.m.f.) and three mordanting methods were used: pre-mordanting, meta-mordanting and post-mordanting. The effects of different mordants and different mordanting methods on the colour parameters were studied and the results are summarized in Table 1.

**Table 1:** The effect of different mordants and mordanting methods on the colour parameters of dyed silk fabrics

Mordants	Mordant dyeing methods	Colour parameter fabric				Images of dyed fabric
		L*	a*	b*	*K*/*S*	
Blank	No mordanting	56.79	10.69	29.39	5.4900	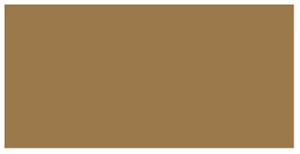
Neodymium trichloride	Pre-mordanting	55.41	10.16	29.20	6.1409	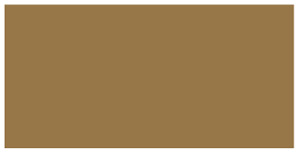
	Meta-mordanting	56.16	10.21	29.43	5.9070	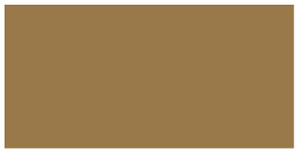
	Post-mordanting	55.66	9.47	27.47	5.3333	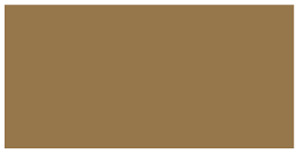
Ferrous sulphate	Pre-mordanting	31.22	2.96	4.76	9.4415	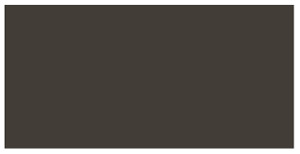
	Meta-mordanting	31.54	2.72	3.47	8.5371	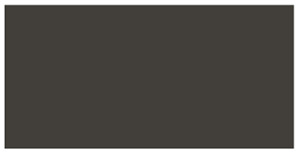
	Post-mordanting	33.22	1.84	5.51	8.3764	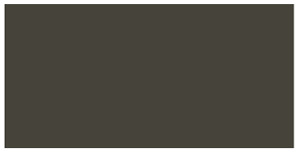
Copper sulphate	Pre-mordanting	52.65	10.30	27.49	6.5955	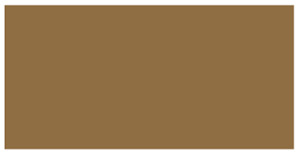
	Meta-mordanting	51.87	10.64	27.73	6.9909	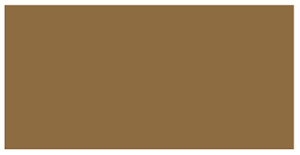
	Post-mordanting	51.74	9.46	25.51	6.2201	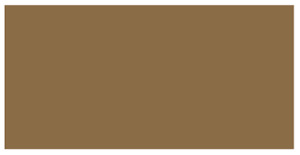
Aluminium sulphate	Pre-mordanting	55.35	10.74	29.45	6.4303	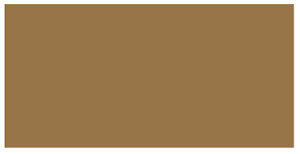
	Meta-mordanting	55.72	10.86	29.47	6.1576	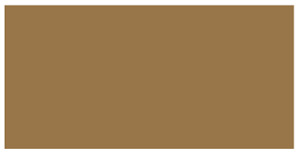
	Post-mordanting	55.29	10.33	27.65	5.6071	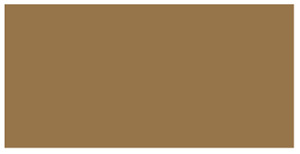
Chromium trichloride	Pre-mordanting	53.11	8.97	28.21	7.2498	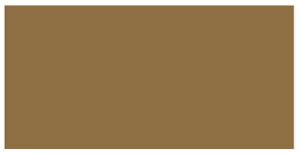
	Meta-mordanting	52.93	8.72	27.49	7.1846	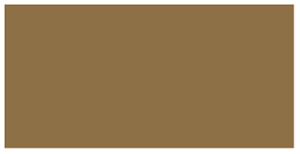
	Post-mordanting	52.11	8.56	25.58	6.3990	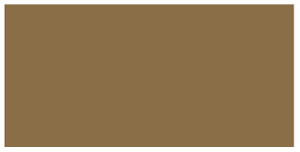
Ferric chloride	Pre-mordanting	31.03	2.77	4.93	9.6213	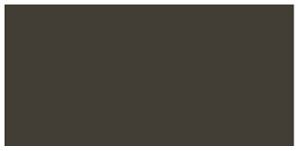
	Meta-mordanting	29.94	2.22	2.54	9.0888	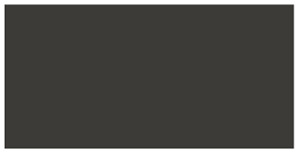
	Post-mordanting	34.23	1.96	6.58	8.2699	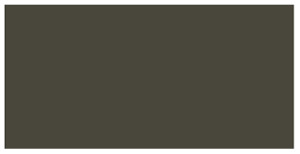

**Fig. 6:**
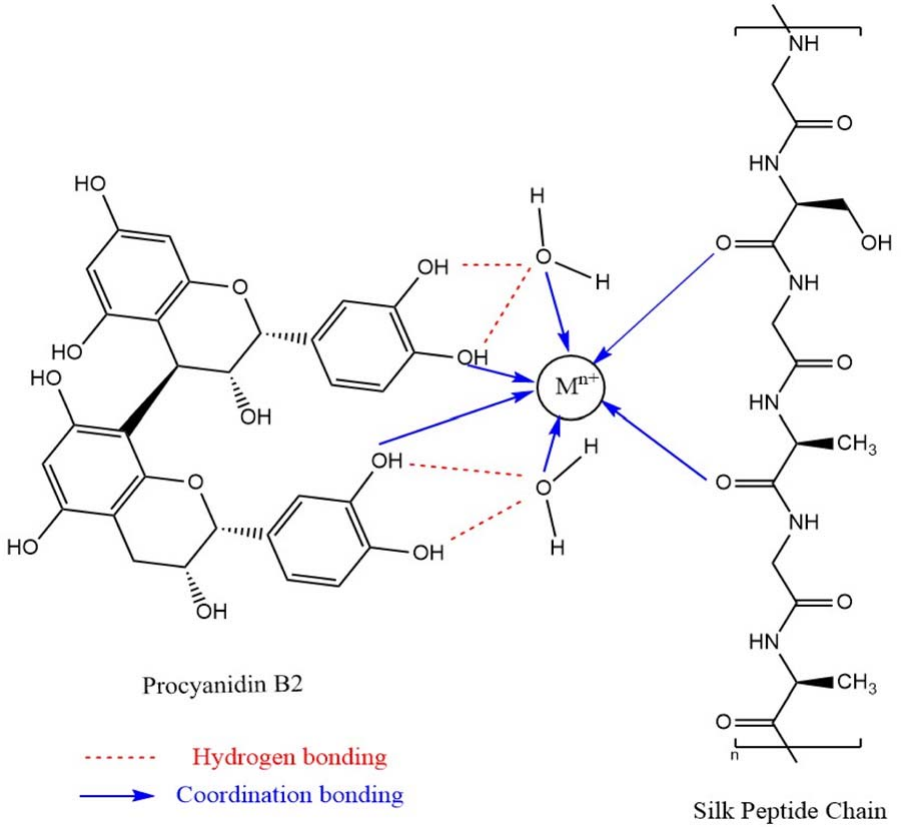
The possible mordanting mechanism.

The results in Table 1 show that the dyed silk fabrics exhibited the same sequence *K*/*S* values in the order of ferric chloride > ferrous sulphate > chromium trichloride > copper sulphate > aluminium sulphate > neodymium trichloride > no mordanting dyed silk fabrics under both pre-mordanting and meta-mordanting conditions, but under post-mordant dyeing conditions, they showed another order: ferrous sulphate > ferric chloride > chromium trichloride > copper sulphate > no mordanting dyed silk fabrics > neodymium trichloride. In most cases, silk fabrics dyed with mordant showed higher *K*/*S* values than those dyed without mordant, and only neodymium trichloride showed lower *K*/*S* values than the no mordanting dyed silk fabrics under post-mordant dyeing conditions. Also, the use of mordant dyeing all resulted in changes in the hue (red–green values a*, yellow–blue values b*) of the dyed silk fabrics. When comparing the three mordant dyeing methods, the *K*/*S* values of the pre-mordant dyeing are generally higher than meta-mordant dyeing and post-mordant dyeing, except for copper sulphate which exhibits higher *K*/*S* values under meta-mordant dyeing conditions. From the perspective of green safety, the residue of metal salts in fabrics and the environment is considered. From the mordanting results, different mordants and mordanting methods have different effects on the colour parameters. This is due to the fact that both natural pigments and silk fibres contain a large number of hydroxyl, amino and carbonyl groups that can provide lone pair electrons. The potential mordant dyeing mechanism with procyanidin B2 was illustrated in Fig. 6. Metal ions could form coordination bonds between the dye and fibre molecules by the lone pair electron bonding to expand the conjugation system of proanthocyanin B2, which leads to the colour change of fabric.^24,25^

#### 3.5.2. Fastness properties

Rubbing fastness and soaping fastness of silk fabrics after mordanting with six metal ions were conducted, the results are shown in Table 2. From the results, it's clear that the colour fastness to rubbing and soaping of the mordant-dyed fabric has been significantly improved, both of which can reach more than 4.0 grade and meet the needs of garment use. The addition of metal ions facilitates the improvement of fabric colour fastness. This is due to the empty p orbitals in the outer layer of metal atoms, which can form pair bonding (coordination bonding) with atoms having lone pairs of electrons (O, N, *etc.*), thus expanding the structural system of dye molecules while enhancing the force between dye molecules and fibres. As a result, the dyestuff won't peel off easily from the fabric and its mechanical colour fastness was improved.

**Table 2:** The effect of different mordants and mordanting methods on the colour fastness of dyed silk fabrics

Mordanting methods	Rubbing fastness			Washing fastness					
	Dry	Wet	Discoloration	Fiber staining					
				Acetate	Cotton	Nylon	Polyester	Acrylics	Wool
Blank	4	3–4	4–5	4–5	5	4	4–5	4	4
Nd^3+^-Pre	4–5	4	5	5	4–5	4–5	5	4–5	4–5
Nd^3+^-Meta	4–5	4	4–5	5	4–5	4–5	5	4–5	4–5
Nd^3+^-Post	4–5	4	5	5	4–5	4–5	5	4–5	4–5
Fe^2+^-Pre	4–5	4	5	5	5	4–5	5	4–5	4–5
Fe^2+^-Meta	4–5	4	4–5	5	5	4–5	5	4–5	4–5
Fe^2+^-Post	4–5	4	5	5	5	4–5	5	4–5	4–5
Cu^2+^-Pre	4–5	4	5	5	5	4–5	5	4–5	4–5
Cu^2+^-Meta	4–5	4	4–5	5	5	4–5	5	4–5	4–5
Cu^2+^-Post	4–5	4	5	5	5	4–5	5	4–5	4–5
Al^3+^-Pre	4–5	4	5	5	5	4–5	5	4–5	4–5
Al^3+^-Meta	4–5	4	4–5	5	5	4–5	5	4–5	4–5
Al^3+^-Post	4–5	4	5	5	5	4–5	5	4–5	4–5
Cr^3+^-Pre	4–5	4	5	5	4–5	4–5	5	4–5	4–5
Cr^3+^-Meta	4–5	4	4–5	5	4–5	4–5	5	4–5	4–5
Cr^3+^-Post	4–5	4	5	5	4–5	4–5	5	4–5	4–5
Fe^3+^-Pre	4–5	4	5	5	5	4–5	5	4–5	4–5
Fe^3+^-Meta	4–5	4	4–5	5	5	4–5	5	4–5	4–5
Fe^3+^-Post	4–5	4	5	5	5	4–5	5	4–5	4–5

### 3.6. Functionalities

#### 3.6.1. UV protection properties

The UV protection properties were then tested and the results are listed in Table 3. From the results, the UV transmittance of the dyed silk fabrics decreased and the UPF values increased. 100 °C dyed silk fabrics showed better UV protection than 80 °C dyed silk fabrics due to the different amounts of dye on the silk fabrics. This result indicates that silk fabrics dyed with camellia husks extract have UV protection and that there is a positive correlation between the amount of dye and UV protection.

**Table 3:** The UV protection properties before and after dyeing

	Undyed silk	80 °C dyed silk	100 °C dyed silk
UVA	16.15	2.25	1.68
UVB	10.15	1.94	1.45
UPF	8.24	49.91	63.40

#### 3.6.2. Antioxidant activities

**Fig. 7:**
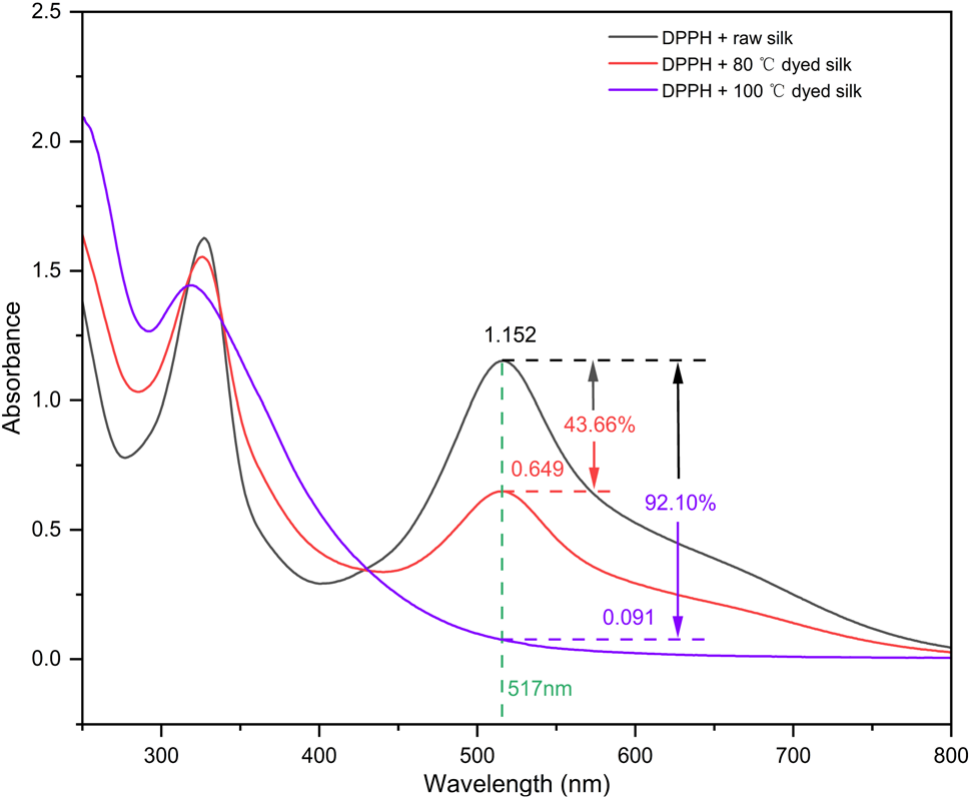
The antioxidant activities of silk fabrics after dyeing.

The antioxidant activity of dyed silk fabric is shown in Fig. 7. 1,1-Diphenyl-2-trinitrophenylhydrazine (DPPH) is an oxidant with a conjugated system that reacts with reducing groups by redox, which is often used to test the antioxidant properties of organic compounds.^26^ From the results, it is clear that the DPPH solution has significant absorption at 517 nm. After adding the raw silk fabric, the change of absorption intensity is small, indicating that the raw silk fabric does not have the group that can react with DPPH by redox without antioxidant properties. After adding the dyed fabric, the absorption peak at 517 nm was obviously weakened. The absorption intensity decreased to 0.061 after dyeing at 100 °C (92.1% decrease). This result indicates that the dyed fabric has excellent antioxidant properties, and its antioxidant capacity is positively correlated with the fabric's colour depth. This is attributed to the fact that the hydroxyl group in procyanidin is capable of redox reaction with DPPH and destroying its conjugate structure, thus making its absorption peak intensity at 517 nm decrease significantly.^26,27^

#### 3.6.3. Antibacterial performance

**Fig. 8:**
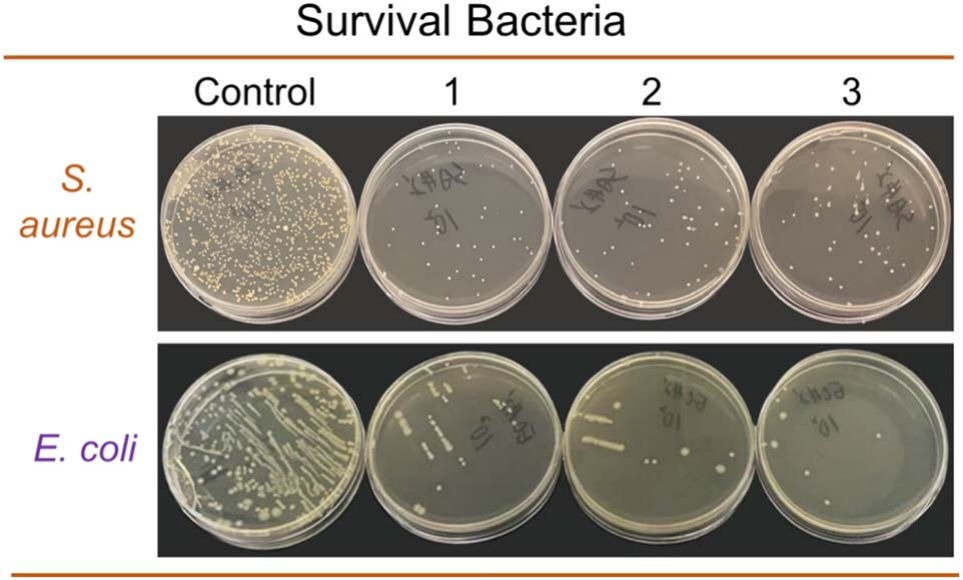
The anti-bacterial properties of dyed fabrics.

In previous reports, procyanidin B2 exhibited some antimicrobial properties when used in metal coatings and medical dressing additions.^16,28^ The antimicrobial properties of silk fabrics dyed with extracted pigments were tested. Three parallel samples with the same strain were tested to compare the antibacterial performance while taking the undyed fabric as a control. The results are presented in Fig. 8. The bacterial inhibition of samples was evaluated by the counting method, which achieved 98.74% and 97.39% against *Escherichia coli* and *Staphylococcus aureus*, respectively. This result is consistent with previous reported results on the antibacterial activity of condensed tannins-like compounds.^29,30^ The antimicrobial mechanism relies mainly on the binding of nonpolar aromatic rings to some cellular proteins through hydrophobic bonds, thus penetrating the hydrophobic region of the lipid bilayer on bacterial cell membranes and eventually leading to the leakage of substances from the membrane.^31^

## 4. Conclusions

In this study, a brown-based natural dye extraction method from camellia husks was proposed and its application properties on silk fabrics were investigated. The optimum dyeing conditions were optimized by single-factor experiments as follows: dyestuff mass 50 g L^−1^, holding time 45 min, bath pH 3.0, holding temperature 100 °C. FTIR and LC-MS analysis of the extracts and dyed fibres showed that the silk fibre was mainly coloured as a dimer or trimer of procyanidin. After mordanting by different methods with six metal mordants, the pre-mordant dyed silk fabric had the best colour yield. The colour fastness to dry and wet rubbing and colour fastness to washing of the dyed silk fabrics were above grade 4. The dyed silk fabrics showed excellent UV resistance, antioxidant activity, antibacterial properties, including the UV resistance index UPF value of 63.4 while the inhibition rate of 98.74% and 97.39% for *S. aureus* and *E. coli*, respectively. The difference in fabric properties was positively correlated with the dye amount on the fabric. The results provide new insights into the re-utilization of camellia husks waste.

## Conflicts of interest

There are no conflicts to declare.

## Supplementary Material

## References

[cit1] Ayele M., Tesfaye T., Alemu D., Limeneh M., Sithole B. (2020). Sustainable Chem. Pharm..

[cit2] Jiang H., Hu X., Zhu J., Wan J., Yao J. (2021). Dyes Pigm..

[cit3] Shahidi S., Moazzenchi B. (2019). Fibers Polym..

[cit4] Fang J., Meng C., Zhang G. (2022). Ind. Crops Prod..

[cit5] Yang Q., Zhang T., He Y., Huang S., Deng X., Han L., Xie C. (2020). Chin. Med..

[cit6] Vankar P., Shukla D. (2011). J. Appl. Polym. Sci..

[cit7] Yang T., Guan J., Chen G., Tang R. (2018). Ind. Crops Prod..

[cit8] Li G., Liu H., Li T., Wang J. (2012). Mater. Sci. Eng., C.

[cit9] Baltova S., Vassileva V. (1998). Polym. Degrad. Stab..

[cit10] Jiang S., Yuen C. W. M., Zhang L., Guo R. H., Choi P. S. R. (2009). Fibers Polym..

[cit11] Li G., Liu H., Zhao H., Gao Y., Wang J., Jiang H., Boughton R. I. (2011). J. Colloid Interface Sci..

[cit12] Li G., Liu H., Li T., Wang J. (2012). Mater. Sci. Eng., C.

[cit13] Zhou Y., Zhang J., Tang R., Zhang J. (2015). Ind. Crops Prod..

[cit14] Di R., Zhang Y., Wu Z., Liu W., Yang C. (2020). J. Mol. Liq..

[cit15] Zhang L., Ho C., Zhou J., Santos J., Armstrong L., Granato D. (2019). Compr. Rev. Food Sci. Food Saf..

[cit16] Huang L., Yang K., Zhao Q., Li H., Wang J., Wu Y. (2022). Bioelectrochemistry.

[cit17] Zheng C., Ma J., Chen J., Ma C., Chen W., Yao M., Chen L. (2019). J. Agric. Food Chem..

[cit18] Guo L., Yang Z., Tang R., Yuan H. (2020). Biomolecules.

[cit19] Sukemi, Pratumyot K., Srisuwannaket C., Niamnont N., Mingvanisha W. (2019). Color. Technol..

[cit20] Ren Y., Fu R., Fang K., Xie R., Hao L., Chen W., Shi Z. (2021). J. Cleaner Prod..

[cit21] Qin Y., Sun Y., Li J., Xie R., Deng Z., Chen H., Li H. (2016). Int. J. Food Prop..

[cit22] Stuart B. , Infrared spectroscopy: fundamentals and applications, John Wiley & Sons, 2004

[cit23] Lin X., Meng L., Chen Y., Qi H., Zhang C. (2021). J. Text. Inst..

[cit24] Ding Y., Freeman H. (2017). Color. Technol..

[cit25] Grifoni D., Roscigno G., Falco E., Vece A., Camilli F., Sabatini F., Fibbi L., Zipoli G. (2020). Fibers Polym..

[cit26] Sun S., Xing T., Tang R. (2013). Ind. Eng. Chem. Res..

[cit27] Luo F., Fei X. (2018). J. Am. Oil Chem. Soc..

[cit28] Zhou L., Pi W., Cheng S., Gu Z., Zhang K., Min T., Zhang W., Du H., Zhang P., Wen Y. (2021). Adv. Funct. Mater..

[cit29] Mayer R., Stecher G., Wuerzner R., Silva R., Sultana T., Trojer L., Feuerstein I., Krieg C., Abel G., Popp M., Bobleter O., Bonn G. (2008). J. Agric. Food Chem..

[cit30] Idowu T., Ogundaini A., Salau A., Obuotor E., Bezabih M., Abegaz B. (2010). Phytochemistry.

[cit31] Xu X., Gong J., Zhang T., Li Z., Zhang J., Wang L., Huang J. (2020). Ind. Crops Prod..

